# Clinicians’ Reasons for Non-Visit-Based, No-Infectious-Diagnosis-Documented Antibiotic Prescribing: A Sequential Mixed-Methods Study

**DOI:** 10.3390/antibiotics14080740

**Published:** 2025-07-23

**Authors:** Tiffany Brown, Adriana Guzman, Ji Young Lee, Michael A. Fischer, Mark W. Friedberg, Jeffrey A. Linder

**Affiliations:** 1 Department of Medicine, Division of General Internal Medicine, Northwestern University Feinberg School of Medicine, Chicago, IL 60611, USA; t-brown@northwestern.edu (T.B.); jlee1@northwestern.edu (J.Y.L.); 2 Oui Therapeutics, New Haven, CT 06511, USA; aguzman@ouitherapeutics.com; 3 Department of Medicine, Section of General Internal Medicine, Boston Medical Center, Boston University Chobanian & Avedisian School of Medicine, Boston, MA 02118, USA; michael.fischer@bmc.org; 4 Blue Cross/Blue Shield of Massachusetts, Boston, MA 02199, USA; mark.friedberg@bcbsma.com; 5 Division of General Internal Medicine, Brigham and Women’s Hospital, Boston, MA 02115, USA

**Keywords:** anti-bacterial agents, antimicrobial stewardship, practice patterns, physicians’, inappropriate prescribing, health services research, quality of healthcare

## Abstract

**Background:** Among all ambulatory antibiotic prescriptions, about 20% are non-visit-based (ordered outside of an in-person clinical encounter), and about 30% are not associated with an infection-related diagnosis code. **Objective/Methods:** To identify the rationale for ambulatory antibiotic prescribing, we queried the electronic health record (EHR) of a single, large health system in the Midwest United States to identify all oral antibiotics prescribed from November 2018 to February 2019 and examined visit, procedure, lab, department, and diagnosis codes. For the remaining antibiotic prescriptions—mostly non-visit-based, no-infectious-diagnosis-documented—we randomly selected and manually reviewed the EHR to identify a prescribing rationale and, if none was present, surveyed prescribers for their rationale. **Results:** During the study period, there were 47,619 antibiotic prescriptions from 1177 clinicians to 41,935 patients, of which 2608 (6%) were eligible non-visit-based, no-infectious-diagnosis-documented. We randomly selected 2298. There was a documented rationale for 2116 (92%) prescriptions. The most common documented reasons—not mutually exclusive—were patient-reported symptoms (71%), persistence of symptoms after initial management (18%), travel (17%), and responding to lab or imaging results (11%). We contacted 160 clinicians who did not document any prescribing rationale in the EHR and received responses from 62 (39%). Clinicians’ stated reasons included upcoming or current patient travel (19%), the antibiotic was for the prescriber’s own family member (19%), or the clinician made a diagnosis but did not document it in the EHR (18%). **Conclusions:** Non-visit-based, no-infectious-diagnosis-documented antibiotic prescriptions were most often in response to patient-reported symptoms, though they also occur for a variety of other reasons, some problematic, like in the absence of documentation or for a family member.

## 1. Introduction

Ambulatory antibiotics account for about 90% of human antibiotic use [1,2]. Antibiotic prescribing is associated with adverse drug events and increases the prevalence of antimicrobial resistant bacteria [3,4,5,6,7,8].

Antibiotic prescribing for outpatients usually occurs during clinic visits and is associated with an infection-related diagnosis code. However, we have found that a large proportion of antibiotics are either non-visit-based or non-infection-diagnosis-related. Depending on the data source, patient population, and methods, 15% to 31% of antibiotic prescriptions are not associated with a synchronous, in-person visit [9,10,11,12], and 8% to 29% are prescribed without an associated appropriate or infection-related diagnosis code [9,10,11,13,14].

Non-visit-based and no-infectious-diagnosis-documented antibiotic prescriptions raise troubling questions about the quality of ambulatory antibiotic prescribing. Many ambulatory antibiotic stewardship interventions are narrowly focused on specific diagnoses at particular locations and may miss a large number of antibiotic prescriptions [15]. Understanding the reasons for prescribing of all antibiotics—especially those prescribed via phone encounters, telemedicine, or otherwise not tied to in-person visits—has the potential to better inform ambulatory antibiotic stewardship programs [16,17,18,19].

Because ambulatory antibiotic prescribing studies generally use claims data or structured electronic health record (EHR) data, they do not assess the clinical context for the prescriptions studied. Examining structured EHR data and unstructured EHR data, as well as surveying prescribers about their otherwise unexplained antibiotic prescriptions, may provide some understanding of why clinicians prescribe non-visit-based and no-infectious-diagnosis-documented antibiotics.

In this sequential explanatory mixed-methods study, our objective was to understand the reason for all antibiotics prescribed in an EHR during a fixed time. To accomplish this, we examined coded EHR data and free-text documentation and, if necessary, surveyed prescribers to understand their reason for non-visit-based, no-infectious-diagnosis-documented antibiotic prescribing.

## 2. Results

### 2.1. Prescription Flow

**Figure 1:**
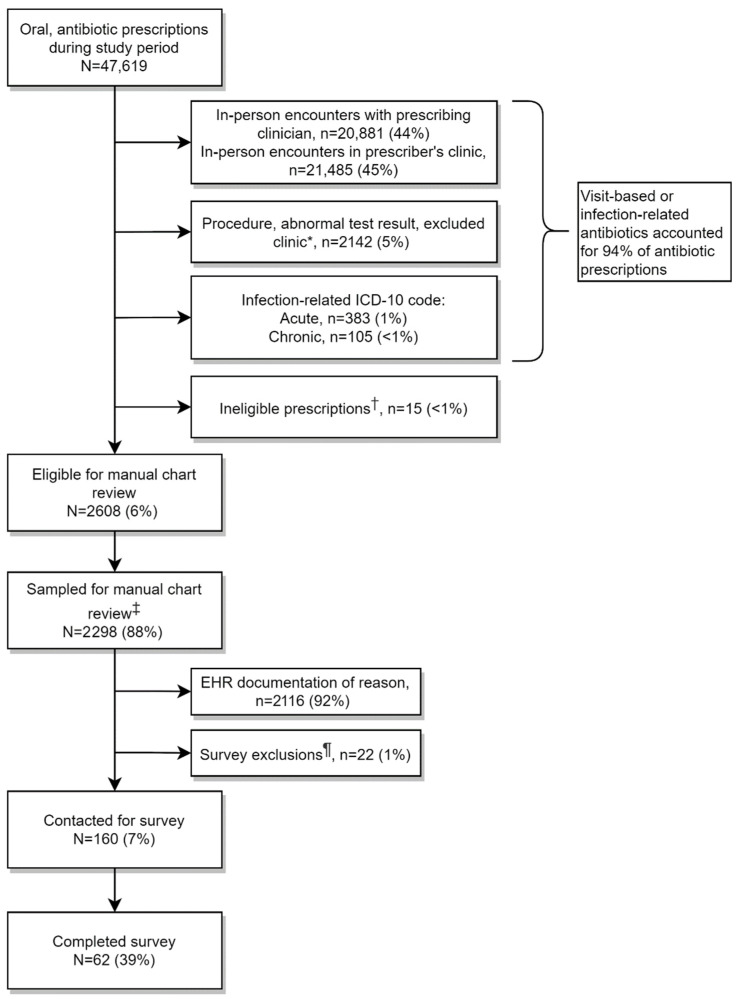
Antibiotic and patient categorization. * Excluded clinics were Reproductive/In Vitro Fertilization Clinics, Travel Medicine, Infusion Clinics, or Hospice. ^†^ Antibiotic prescriptions were ineligible for manual chart review because the prescribing clinician reached the contact limit of three separate prescriptions, the clinician refused to participate, orders were pending or for deceased patients, or we were unable to differentiate between specific antibiotics due to multiple orders. ^‡^ These randomly selected 2298 antibiotic prescriptions were from 500 physicians to 2201 patients. ^¶^ Survey exclusions included in-person encounters between the order date and chart review, the patient receiving palliative care at the time of chart review, and the absence of secure clinician contact information to securely administer the survey.

During the study period, there were 47,619 antibiotic prescriptions ordered by 1177 clinicians for 41,935 patients (Figure 1).

Most of the prescriptions were ordered within in-person clinician encounters at the prescriber’s clinic (89%). Non-visit-based antibiotics prescribed by clinicians in surgical departments, within 72 h of an abnormal urine culture/urinalysis, or by clinicians in excluded clinics accounted for 5% of antibiotics. One percent of non-visit-based antibiotic prescriptions were associated with an acute or chronic infection-related ICD-10 code. In all, 94% of antibiotic prescriptions were visit-based or infection-related. A small number (*n* = 15) of non-visit-based, non-infection-diagnosis-related antibiotics were excluded as ineligible for chart review for other reasons. This left 2608 antibiotic non-visit-based, no-infectious-diagnosis-documented antibiotic prescriptions eligible for manual chart review, of which we randomly selected 2298.

### 2.2. Prescription, Patient, and Clinician Characteristics

Patients were 63% female with a mean age of 42 years (SD, 23), and most (44%) had private insurance (Table 1). Compared to all patients, patients who had non-visit-based, no-infectious-diagnosis-documented antibiotic prescriptions and were randomly selected for manual chart review tended to be older, have Medicare insurance, receive more medications, and have a primary care visit.

Clinicians were 61% female and 79% physicians. Among prescribing physicians, 42% were in primary care and 52% were in other medical specialties. Compared to all clinicians, clinicians who prescribed non-visit-based, no-infectious-diagnosis-documented antibiotics tended to be in primary care and be more clinically active (i.e., have a greater clinical full-time equivalent).

The most common antibiotic classes were penicillins (36%), macrolides (25%), and cephalosporins (15%). There were no clinically meaningful differences between clinicians or patients who prescribed or received, respectively, any antibiotics or WHO-AWaRe “Watch” antibiotics (antibiotics of particular concern in promoting antibiotic resistance and as a target for antibiotic stewardship) [20].

### 2.3. Manual Chart Review

Of the 2298 antibiotic prescriptions manually reviewed, we found a documented reason for the antibiotic order for 2116 (92%; Table 2). The most common explanations (not mutually exclusive) were patient-reported symptoms (71%) and persistence of symptoms after initial management (18%). Among patient-reported symptoms, the most common types were respiratory and urinary. There were no marked differences between the use of any antibiotic or the WHO-AWaRe “Watch” antibiotics.

Many of the antibiotic orders (37%) were associated with more than one encounter within the record (e.g., the patient sent a secure message one day and two days later telephoned, with both encounters related to the antibiotic order), and 34% of these associated encounters were with a clinician other than the prescribing clinician (e.g., a patient originally spoke with one doctor, and four days later, when a patient called to request an antibiotic due to worsening symptoms, the first doctor was not available, so the patient spoke with a second doctor in same clinic as the first). For 12% of the antibiotics manually reviewed, the patient and clinician dyad had a subsequent encounter (of any type) within 7 days of the order.

Descriptions of each of the primary explanation categories with examples of EHR narrative notes are provided in Table 3. Some explanations, not well-captured in the EHR, involved multiple clinicians coordinating patient care (e.g., a patient was referred by a hematologist to a primary care clinician to discuss receiving an antibiotic).

### 2.4. Clinician Survey Data

We contacted clinicians regarding *n* = 160 antibiotic orders and received responses from 47 clinicians regarding 62 (response rate, 39%) antibiotic prescriptions. Among respondent clinicians were 24 (51%) women, 40 (85%) physicians. Responding physicians had graduated from medical school a mean of 29 (standard deviation, 10) years prior, and 33 (83%) were primary care physicians. Although our goal was to survey clinicians within 7 days of the order date, some clinicians were contacted up to 23 days after the order date due to capacity constraints (the majority were contacted within about 8 days). Clinician participants were 51% female and 74% primary care. There were no statistically significant differences between responders and non-responders in gender and specialty. The most commonly prescribed antibiotics were azithromycin (44%), amoxicillin (14%), amoxicillin–clavulanate (8%), ciprofloxacin (8%), and cefdinir (3%).

Themes from survey data are summarized in Table 4 with illustrative quotes. The most common codes for clinicians’ primary reasons for prescribing non-visit-based antibiotics were patient travel (19% of responses), order was for a family member (19%), and the clinician made a diagnosis but did not document it within the EHR (18%). Half (50%) of the responses had an applicable secondary code, the most common of which were “diagnosis made but not documented” and “patient-reported symptoms.”

Patient travel included current or planned domestic or international travel. Some cases consisted of patients experiencing symptoms at the time of request, and other patients with planned travel requested antibiotics ahead of travel in case they were needed while away. Antibiotic prescriptions for family members appeared to be mainly for convenience (i.e., the prescriber and family member did not want to seek care in some other way).

### 2.5. Appropriateness of Survey-Described Antibiotic Prescribing

Of the 62 survey responses, 21 (34%) were likely appropriate (e.g., otitis media in the child of a physician’s practice partner, seen at home and mentioned a specific diagnosis), 17 (27%) were inappropriate (e.g., antibiotics in response to symptoms, domestic travel, or failure to document), 14 (23%) were possibly inappropriate (e.g., treating a family member), and 10 (16%) were unable to be determined.

## 3. Discussion

In this sequential mixed-methods study, we found that 94% of antibiotic orders were associated with in-person encounters or were infection-related, and only 6% were non-visit-based and non-infection-diagnosis-related. Concerningly, roughly half of the antibiotics were WHO-AWaRe “Watch” antibiotics, although there did not appear to be clinically important differences between the clinicians, patients, and reasons between all antibiotic use and “Watch” antibiotic use.

Based on chart review of the remaining prescriptions, we were able to identify a reason for antibiotic prescribing for 92% (*n* = 2116) of cases, most often as a response to new patient symptoms, persistence of symptoms after initial management, travel, and responding to lab or imaging results. In surveying the remainder, clinicians mentioned prescribing antibiotics for travel, for their own family members, failing to document a reason for the prescription in the EHR, or prescribing antibiotics in the absence of an evaluation. Many of these prescriptions could be associated with poor medical care.

Methodological differences explain the higher proportions of non-visit-based (15% to 31%) and no-infectious-diagnosis-documented (8% to 29%) prescribing in prior studies [9,10,11,12,13,14]. First, this analysis was focused on prescribing with the potential to be of particular concern: antibiotics that were *both* non-visit-based *and* non-infection-diagnosis-related. Other studies could not ascertain the site of prescribing or examined prescriber and diagnosis association separately. Second, our data source was the EHR from a single health system in which antibiotic prescribing might be more easily linked to a reason than claims data which may have different origins of data for prescribing, site-of-care, and diagnosis. Third, in the present study, we allowed for more other acceptable reasons for antibiotic prescribing, notably visits to certain clinics, temporal association with abnormal urinalyses, chronic infection diagnoses within 6 months, and surgical procedures within 31 days.

Although we were not mainly assessing appropriateness, some apparent non-visit-based, no-infectious-diagnosis-documented prescribing seems appropriate. As examples, antibiotic prescribing in response to a new laboratory or imaging result could be reasonable. Prescribing antibiotics to women with a history of urinary tract infections, who have typical symptoms without symptoms of pyelonephritis, is acceptable [21]. Similarly, clinicians in different clinics discussing a patient and coordinating care could represent excellent communication and convenient treatment.

On the other hand, many of these reasons seem suspect or clearly inappropriate. Prescribing antibiotics for a new or ongoing symptom without an evaluation or diagnosis may result in poor medical care. Guidelines and recommendations about prescribing antibiotics for travel are, at best, confusing: antibiotics are effective but should not be used, except for moderate or severe diarrhea, which are vaguely differentiated from mild diarrhea [22]. Writing a prescription for a family member or clinic staff member is strongly discouraged except in limited circumstances (e.g., emergency situations where there is no other qualified physician available and only for short-term, minor problems) [23]. Writing a prescription without documentation poses a quality assessment and stewardship problem. In addition, antibiotic prescribing to patients without documentation or to patients who are out of state might also carry medicolegal risks; antibiotics are one of the most common medication types associated with litigation [24].

This study has limitations. First, as a pragmatic assessment of routine care, our study was documentation-dependent, relied on diagnosis codes, narrative notes from clinicians, and manual search of this documentation to determine reasons for antibiotic prescribing; the reason was sometimes minimal or not present. Second, the response rate to our survey was only 39%. We found no significant differences between responders and non-responders in gender and specialty. One would expect non-responders to differ from responders, with responders potentially being more conscientious prescribers and more likely to give socially desirable responses; other true reasons might be more problematic. Third, this assessment was limited to a single health system; other health systems may have organizational differences (e.g., varying EHR documentation requirements or health system prescribing guidelines) that could affect the results. Fourth, the data have aged but are still valid—the analysis was performed prior to the COVID-19 pandemic, which resulted in decreases in ambulatory visits, especially for respiratory symptoms, increases in telemedicine visits (uncommon at the time of this study), and changes in antibiotic prescribing, which may be related to an increase in non-visit-based antibiotic prescribing [25,26,27,28]. Fifth, as the study was conducted in a typical viral respiratory season, this could have led patients to actively seek to avoid in-person exposures in clinics; assessments at different times of year may differ. Sixth, this study would not capture antibiotic prescribing outside of the EHR (e.g., by a clinician directly calling a pharmacy). Seventh, this was a study of clinician antibiotic prescribing behavior and not of patient antibiotic exposure.

Despite these limitations, these findings identify several issues that may affect contemporary practice, research, and antibiotic stewardship initiatives. First, researchers studying or antibiotic stewards addressing antibiotic prescribing for specific conditions, like respiratory diagnoses, or requiring certain conditions when defining a cohort, like an in-person visit, should realize they are missing antibiotic prescribing [15]. If the goal is to measure or reduce overall antibiotic use, then researchers or antibiotic stewardship teams should measure overall antibiotic use [28,29]. Second, stewardship programs using survey or claims data to assess antibiotic prescribing should realize that the proportion of antibiotic prescriptions that appear to be non-visit-based and no-infectious-diagnosis-documented may appear so because of the complexity of medical care (e.g., testing ordered in one clinic, followed up in another), limitations of documentation, data capture, and data transmittal. Third, to address these data limitations, health systems and clinicians should consider requiring reasons for antibiotic prescribing, something pharmacists have been requesting for decades [29,30].

From an antibiotic stewardship perspective, prescribing inappropriate antibiotics and failing to document an appropriate reason for an antibiotic prescription are functionally the same. Requiring clinicians to provide a diagnosis or rationale for antibiotic prescribing reduces inappropriate antibiotic prescribing and would facilitate ongoing quality improvement and antibiotic stewardship [31]. Other effective antibiotic stewardship interventions include communication training, requiring clinicians to publicly commit to appropriate antibiotic prescribing, requiring clinicians to justify seemingly inappropriate prescriptions, and peer comparison interventions [31,32,33,34,35]. At the same time, antibiotic stewardship programs, given time, personnel, or budget constraints, should consider whether it is worth the effort to address the 20% of all antibiotic prescriptions that are not associated with a visit [9,10,11,12], the 30% not associated with an infection-related diagnosis code [35], or, most specifically, the 6% of antibiotic prescribing that is *both* non-visit-based and no-infectious-diagnosis-documented. Additionally, this and other retrospective studies are challenged to identify complications, visits, or hospitalizations that might have been prevented by poorly documented antibiotic prescribing.

In conclusion, we found that a reason for non-visit-based, no-infectious-diagnosis-documented antibiotic prescriptions is often available in non-coded, free-text portions of the EHR. These antibiotics are most often prescribed in response to new or ongoing patient symptoms but also for travel and response to lab or imaging results. Of antibiotic prescriptions without a documented reason, in surveying clinicians, we found that most of the remaining prescriptions were for travel, a family member, or simply due to failure to document the reason for the prescription. These findings should inform the development and evaluation of interventions to improve the quality of outpatient antibiotic prescribing, including antibiotic prescribing that is undocumented and seemingly problematic.

## 4. Materials and Methods

### 4.1. Setting and Overview

We conducted a sequential mixed-methods study combining the review of EHR data with a survey within Northwestern Medicine, a large, academic health system in the Chicago area (Illinois, USA) [36]. The entire system uses a single EHR (Epic Systems, Verona, WI, USA). Data are stored in the Northwestern Medicine Enterprise Data Warehouse and are updated nightly [37]. The Data Warehouse currently stores observations on more than 6.6 million distinct patients. The EHR organizes units of work into “encounters” of differing types such as clinic visits, telephone calls, patient portal messages, and refills, among others. An encounter is an organizational attempt to collect all aspects of a care episode (e.g., communication with the patient and within the healthcare team, documentation, ordering, and billing) within a single unit of work. All prescriptions are generated within a defined encounter type in the EHR.

We identified all antibiotic prescriptions for outpatients between November 2018 and February 2019 to examine antibiotic prescribing during a typical viral respiratory infection season. We used visit, procedure, lab, department, and diagnosis codes to identify antibiotic prescribing with a potential rationale. We randomly sampled the remaining antibiotic prescriptions without a rationale—mostly non-visit-based, no-infectious-diagnosis-documented antibiotic prescriptions—to see if there was a rationale documented in the EHR. If, after manual chart review, we still could not find a rationale for antibiotic prescribing, we planned to survey the prescribing clinicians within 7 days of the prescription. For all but the last step in our evaluation, our goal was not to assess the *appropriateness* of antibiotic prescriptions. Rather, we sought to identify a *reason*, or rationale, for all ambulatory antibiotic prescribing.

### 4.2. Data Extraction

We queried the Data Warehouse weekly from November 2018 to February 2019. The query was validated by manual chart review prior to study initiation. We identified all oral, non-absorbable antibiotics prescribed in ambulatory clinics. Although prescribers could theoretically “call in” antibiotic prescriptions over the phone, the common practice was that all antibiotic orders were generated and sent using the EHR. We extracted antibiotic (e.g., antibiotic class), patient, and prescribing clinician data. Because of their importance in promoting antibiotic resistance and as a target for antibiotic stewardship, we examined prescriptions of the WHO-AWaRe (World Health Organization “Access–Watch–Reserve”) “Watch” antibiotics [37].

We calculated clinician full-time equivalents (cFTEs) by counting the number of half-day clinic sessions (separated at 12 noon) where clinicians had at least 3 patient in-person encounters for each half day of employment.

### 4.3. Identifiable Reasons for Antibiotic Prescribing

We used visit, procedure, lab, department, and diagnosis codes to identify antibiotic prescribing reasons. We defined visit-based antibiotic prescriptions as antibiotics ordered the same day or within 7 days of a clinical encounter with the prescriber or with a clinician in the prescriber’s clinic. We defined procedure-related antibiotic prescriptions as antibiotics prescribed by clinicians in surgical departments to patients with a scheduled or completed procedure or surgery within 31 days before or after the antibiotic prescription. Our presumption was that these prescriptions were for the condition related to surgery, pre-operative prophylaxis, or post-operative infection.

We defined antibiotic prescriptions within 72 h of an abnormal urinalysis or urine culture as for treatment of a urinary tract infection. We defined antibiotic prescriptions from clinicians in Travel Medicine, Reproductive/In Vitro Fertilization, Infusion, or Hospice Departments as related to care in those departments and considered these clinics “excluded.” Thus, non-visit-based antibiotics were those prescribed at a non-excluded clinic in the absence of an in-person encounter, a procedure, or an abnormal result.

For the remaining antibiotic prescriptions, we used a published classification in which all 94,249 International Classification of Diseases, 10th Edition (ICD-10) codes are defined as “always”, “sometimes”, or “never” justifying antibiotic prescribing [13]. We defined acute, infection-related antibiotics as antibiotic prescriptions associated with “always” or “sometimes” antibiotic-appropriate diagnosis codes. We defined chronic, infection-related antibiotics as those prescribed within 6 months of one of the 690 ICD-10 chronic infection or chronic condition codes (e.g., chronic obstructive pulmonary disease, acne, and others) [9,10]. Thus, no-infectious-diagnosis-documented antibiotics were those prescribed in the absence of an “always” antibiotic-appropriate, a “sometimes” antibiotic-appropriate, or a “chronic” infection-related antibiotic code.

### 4.4. Manual Chart Review Form

To abstract information about antibiotic prescriptions for which there was no identifiable rationale based on structured data from the EHR, we developed a chart abstraction form (Appendix A) and piloted it prior to study initiation. We created categories of reasons for antibiotic prescribing that we anticipated clinicians may document within unstructured EHR fields (e.g., visit, telephone, or electronic communication notes) such as patient-reported symptoms or recently resulted labs. Chart reviewers were not evaluating the clinical appropriateness of a prescribing explanation but rather looking for the presence/absence of a reason for the antibiotic order. All data were collected using Research Electronic Data Capture (REDCap), and the chart abstraction form was pre-populated with identifying case details, such as patient name, date of birth, encounter date/type, prescriber name, prescription date, and associated diagnosis, if any [38].

Prior to the start of the study, chart reviewers (AG and TB) independently reviewed the same *n* = 50 cases and compared agreement for data capture and presence of an antibiotic prescription rationale. While they agreed on whether a rationale for the antibiotic prescription was or was not present for all 50 cases, minor adjustments were made to the chart abstraction form to improve clarity and consistency.

The abstraction form included discrete variables indicating yes/no to whether a rationale existed for the antibiotic prescription and an ability to select multiple reasons for the antibiotic prescription (i.e., the rationales were not mutually exclusive). The abstraction form also included space to extract the section of the EHR visit note that mentioned the antibiotic order verbatim in a free-text field when more detailed information was available beyond that captured by the discrete variables.

For antibiotic orders with a rationale found upon manual chart review, the abstraction form included fields for documentation of additional encounters, of any type, associated with the antibiotic order, the date they occurred, and if they were with the prescribing clinician.

### 4.5. Selection for Chart Review

Due to staff time constraints, we anticipated not being able to follow up on all remaining antibiotic prescriptions without an identifiable rationale in a timely manner. Thus, we randomly sampled the remaining antibiotic prescriptions for manual chart review to determine whether there was an identifiable rationale documented. Given that we had two reviewers and wanted to review all antibiotic prescriptions within one week of prescribing, we estimated 200 chart reviews per week over the course of three months of manual chart review and so randomly selected about 200 charts per week (with winter holidays and staff vacation, we were able to review almost 2300 charts; in retrospect, the total number of charts only slightly exceeded this limit). We excluded antibiotic orders that were subject to manual chart review from clinicians who had previously been contacted three times (to reduce participant burden) or refused to participate. Any antibiotic orders pending (i.e., not actually prescribed) or for deceased patients at time of review were excluded from manual chart review. Although rare, we excluded orders from manual chart review where we were unable to differentiate between specific antibiotics due to multiple orders.

### 4.6. Clinician Survey

For antibiotic prescriptions without an identifiable rationale using coded data or through chart review, we aimed to survey the prescribing clinician within 7 days of the antibiotic order. We did not survey clinicians for whom we did not have secure contact information necessary to administer the survey. We did not survey clinicians if the patient was receiving palliative care or the clinician/patient dyad had an in-person encounter between the date of the query and the date of chart review.

We contacted eligible clinicians via a secure email from a study-specific email address that a single staff member (AG) had credentials to access to protect clinician confidentiality. The initial email contained details about the antibiotic prescription and stated “We are asking you to reply to this email with (a) a brief explanation for why you prescribed this antibiotic, and (b) why you prescribed this antibiotic without seeing the patient in the office.” All survey contacts and responses were recorded and stored in REDCap. Clinicians received up to three reminders via email or phone to complete the survey if they failed to respond to prior contacts.

### 4.7. Data Analysis

We used descriptive statistics to describe the cohort of patients and clinicians. As patients and clinicians could enter the cohort more than once, we used their descriptive data at first entry into the cohort. At the end of data collection, we collapsed some prescription rationale categories on the abstraction form into broader groups (e.g., cough and sinus variables were collapsed into the “respiratory” symptoms category). Therefore, we also reviewed “Other, specify below” category responses for primary rationales and reallocated responses to better fit the final prescription rationale categories, if possible (e.g., shortness of breath moved from “Other, specify below” to “Respiratory” symptoms category). All quantitative analyses were performed using SAS v9.4 (SAS Institute Inc., Cary, NC, USA). As this study sought to describe the apparent reasons for ambulatory antibiotic prescribing and we did not make comparisons between groups, sample size calculations for number of antibiotic prescriptions or clinicians were not necessary.

Qualitative data from clinician survey responses were fully de-identified and analyzed using constant comparative analysis [39]. All study team members reviewed all clinician survey responses and met multiple times to iteratively identify and refine dominant themes. Two coders (AG and TB) then independently coded each survey response. Each response was a row in the dataset and could have more than one code if applicable. All coding discrepancies were resolved via discussion. For this last step, one clinician coder (JAL) classified each antibiotic order using generally accepted antibiotic prescribing guidelines to determine clinical appropriateness based on clinician response.

## Figures and Tables

**Table 1:** Patient and clinician characteristics.

Patient Characteristics	All Patients (n = 41,935) ^1^	Patients with Manual Chart Review	
		All Antibiotics (n = 2201) ^2^	“Watch” Antibiotics (n = 1208) ^3^
Age in years, mean (SD)	42 (23)	52 (20)	54 (19)
Female, n (%)	26,519 (63)	1490 (68)	749 (62)
Ethnicity—Hispanic or Latino, n (%)	3208 (8)	98 (4)	40 (3)
Race, n (%)			
Asian	1623 (4)	53 (2)	33 (3)
Black	2728 (7)	124 (6)	53 (4)
White	32,681 (78)	1805 (82)	1000 (83)
Other/Unknown	4903 (12)	219 (10)	122 (10)
Insurance, n (%)			
Private Medicaid Medicare Self-pay/other	18,288 (44) 15,482 (37) 7422 (18) 743 (2)	838 (38) 701 (32) 607 (28) 55 (3)	469 (39) 354 (29) 353 (29) 32 (3)
Comorbidities, median (IQR)	0 (0, 1)	0 (0, 2)	0 (0, 2)
# of other prescriptions, median (IQR)	2 (0, 5)	4 (2, 7)	4 (2, 8)
# of Physician visits in period, median (IQR)	4 (1, 8)	5 (2, 9)	4 (2, 9)
# of ED visits in period, n (%)			
0 1 2+	41,420 (99) 457 (1) 58 (<1)	2165 (98) 35 (2) 1 (<1)	1187 (98) 20 (2) 1 (<1)
# of hospitalizations in period, n (%)			
0 1 2+	38,071 (91) 2585 (6) 1279 (3)	1967 (89) 165 (8) 69 (3)	1068 (88) 99 (8) 41 (3)
Primary care clinician listed, n (%)	36,108 (86)	2043 (93)	1136 (94)
**Clinician Characteristics**	**All Prescribers** **(n = n = 1177) ^4^**	**Clinicians with Manual Chart Review**	
		**All Antibiotics** **(n = 500) ^5^**	**“Watch” Antibiotics** **(n = 326) ^6^**
Female, n (%)	721 (61)	310 (62)	187 (57)
Clinician type, n (%)			
Physician APN/NP/midwife ^7^ Physician assistant	928 (79) 161 (14) 88 (8)	412 (82) 54 (11) 34 (7)	284 (87) 23 (7) 19 (6)
**Limited to Physicians**	**N = 928**	**N = 412**	**N = 284**
Specialty, n (%)			
Primary care Medical Surgical Other	392 (42) 485 (52) 40 (4) 11 (1)	242 (59) 150 (36) 16 (4) 4 (1)	191 (67) 82 (29) 8 (3) 3 (1)
Clinical full-time equivalent, n (%)			
≤25% 26–50% 51–75% 76–100%	351 (38) 147 (16) 208 (22) 222 (24)	73 (18) 66 (16) 116 (28) 157 (38)	49 (17) 33 (12) 72 (25) 130 (46)
Years since medical school graduation, mean (SD)	22 (11) ^8^	23 (11) ^9^	23 (11) ^10^

^1^ During the study period, there were 47,619 antibiotic prescriptions to 41,935 unique patients. 2 Among chart review prescriptions, there were 2298 antibiotics prescribed to 2201 unique patients. Patients with manual chart review prescriptions are a subset of All Patients and are presented to show clinical differences between groups. ^3^ Among chart review prescriptions for WHO-AWaRe “Watch” antibiotics, there were 1252 antibiotics prescribed to 1208 unique patients. Patients with manual chart review who received WHO-AWaRe “Watch” antibiotics are a subset of All Antibiotics and are presented to show clinical differences between groups. ^4^ During the study period, 1177 prescribers generated 47,619 antibiotic prescriptions. 5 Among chart review prescriptions, there were 500 unique prescribers for the 2298 antibiotic prescriptions. Clinicians with manual chart review prescriptions are a subset of All Prescribers and are presented to show clinical differences between groups. ^6^ Among chart review prescriptions for WHO-AWaRe “Watch” antibiotic prescriptions, there were 326 unique prescribers for the 1252 “Watch” antibiotic prescriptions. ^7^ APN is Advance Practice Nurse. NP is Nurse Practitioner. ^8^ N = 774 due to missing values. ^9^ N = 394 due to missing values. ^10^ N = 277 due to missing values.

**Table 2:** Chart review—documented reasons for non-visit-based, no-infectious-diagnosis-documented antibiotic prescriptions.

Reason	All Antibiotics (n = 2116) ^1^	%	“Watch” Antibiotics (n = 1146) ^2^	%
Patient-reported symptoms	1500	71	790	69
Respiratory	984		658	
Urinary	306		83	
Skin/soft tissue	140		14	
Fever	56		40	
GI	53		29	
Dental	7		1	
Other symptoms	4		0	
Persistence of symptoms after initial management	376	18	191	17
Travel	260	12	225	20
Lab/imaging results	242	11	75	7
Evaluated in clinic	195	9	111	10
Refill request	42	2	8	1
Peri-procedural prophylaxis	15	1	5	<1
Other reasons ^3^	166	8	81	7

^1^ We found a documented reason for prescribing for 2116 of 2298 (92%) sampled non-visit-based, no-infectious-diagnosis-documented antibiotic prescriptions. Reasons are not mutually exclusive. The most commonly prescribed antibiotics were azithromycin (33%), amoxicillin–clavulanate (12%), ciprofloxacin (9%), nitrofurantoin (8%), and amoxicillin (7%). ^2^ The most commonly prescribed WHO-AWaRe “Watch” antibiotics were azithromycin (60%), ciprofloxacin (17%), levofloxacin (6%), cefdinir (6%), and cefuroxime (5%). ^3^ The most common “Other” reasons were drug reactions, medication switches, and pharmacy and insurance issues.

**Table 3:** Chart review: main categories of documented rationales for non-visit-based, no-infectious-diagnosis-documented antibiotic orders.

Category	Description	Illustrative Extracted Explanation from EHR
Patient-reported symptoms	Patient initiates contact with clinician and reports symptoms/seeks treatment.	“Patient says she is having annual sinus congestion symptoms, going on vacation and does not want to go in for office visit” [ID 84] “Has had LLE extremity pain and swelling × 3 days. Has pain in her R leg. No fevers.” [ID 223]
Lab/imaging results	Clinician receives lab or imaging results and places order in response.	“Urine culture shows urinary infection with a bacteria called Klebsiella… It will respond more fully to cefixime once daily instead of the nitrofurantoin she already started.” [ID 2034] “Pap did show BV and will need treatment to prevent preterm contractions.” [ID 1793]
Evaluated in clinic	Patient seen by another clinician or by the prescribing clinician but not documented as an in-person encounter	“Pt had office visit in hematology where she presented with ongoing URI symptoms including sore throat, cough, adenopathy, and today low grade fever. Advised she follow up with her PCP again to discuss if she needs additional course of antibiotics.” [PCP then prescribes antibiotics via orders only.] [ID 1895]
Peri-procedural prophylaxis	Patient has recently completed or upcoming scheduled procedure	“Scheduled for Mohs surgery…abx pre op due to mtx and pred. Immunocompromised.” [ID 2457] “Patient is calling requesting amoxicillin before her dentist appointment.” [ID 1297]
Travel	Patient reports current or planned domestic or international travel. Includes both requests due to symptoms and “just in case” requests.	“[Patient] called from out of town complaining of three weeks of sinus pain worsening…” [ID 350] “Patient called in, states is leaving this evening [OUT OF COUNTRY]. Just had a pedicure completed and foot was cut and does not want to seek treatment out there if an infection develops. Patient asking if a script can be called in for antibiotic just in case.” [ID 863] “I am going to Mexico on Friday, to a resort where I have never gotten sick before, but just in case can you give me a prescription for the antibiotic I should take…” [ID 243]
Refill request	Patient requests refill or clinician uses “refill” order mechanism to prescribe.	“Spoke to pt and he said you usually send him refill with antibiotic.” [ID 1700] [From patient email]: “I’m having diverticulitis symptoms again and am wondering if you can call in a refill or prescription for Ciprofloxin which is what you’ve prescribed for past flare ups.” [ID 1896]
Persistence of symptoms after initial management	Follow-up about a condition/symptom that patient/clinician dyad has previously discussed.	“Pt has finished 10 days of abx for sinus infection. Was feeling better while on abx, but now sx are coming back. Pt says he usually has to do a double dose of abx for sinus this time of year.” [ID 937] [From RN]: “Pt finished her antibiotics ordered [15 days ago]. Bactrim DS for 5 days. She still feels like ‘something isn’t right’ and discomfort.” [From MD]: “UCx resistant to bactrim. Treat with cipro.” [ID 1282]

**Table 4:** Clinician survey: themes explaining non-visit-based, no-infectious-diagnosis-documented antibiotic orders.

Theme	Description	Illustrative Clinician Quote
Clinic Staff	Antibiotic prescribed for staff in prescriber’s clinic.	“[Patient] is one of our staff at the office. I gave her a zpak for an upper respiratory infection on the side without an office visit.” [ID 628]
Continuation of previous condition	Follow-up about a condition/symptom that patient/clinician dyad has previously discussed.	“[Patient] was seen 10 days earlier with bronchitis—he was starting to improve 7 days after starting Zpak when symptoms returned on day 8 a change in antibiotics was warranted as he was having the same symptoms as he presented with a week prior” [ID 1420]
Diagnosis made, but not documented in EHR	Clinician referenced patient diagnosis but did not document it in EHR.	“Sinusitis for two weeks.” [ID 620] “He had recurrent diverticulitis over the holidays…” [ID 1263]
Evaluated in clinic	Patient evaluated in clinic without EHR documented encounter.	“The patient brought his kid in. Had strep throat. [Patient] said when he was leaving ‘I have the same symptoms’. Looked in his throat, he had a red exudative throat. Patient was put on an antibiotic. I was slammed that day, did not have time to see him, nor did I have time to put anything on his chart.” [ID 1036]
Evaluated outside of clinic	Patient evaluated by clinician outside of clinic (e.g., patient’s home).	“Did not put my notes in yet but he was a home visit.” [ID 46] “I did see her on my weekly dialysis rounds and sent a urinalysis and urine culture prior to prescribing antibiotics.” [ID 2295]
Family	Patient related to prescribing clinician.	“He is my son and I examined him at home.” [ID 2138] “My wife has had repeated UTIs that have responded to cipro. We were [OUT OF STATE] when she began to have the same sx and I prescribed the Cipro for her.” [ID 1874]
Infection Exposure	Patient came in contact with contagious infection.	“I saw the sib that day with strep. Rapid test positive in my office. This sib was at home with same symptoms—fever, ST no uri symptoms so I told mom I would treat her too. This is a single mom, 3 kids, works full time—my discretion—my decision to treat sib.” [ID 825]
Lab/imaging results	Clinician receives lab or imaging results and places order in response.	“This UA was ordered by cardiology and patient was scheduled for an angiogram and we being the PCP they wanted us to treat her UTI prior to the angiogram. The UA was done by them that’s why there was no documentation in her file by her cardiologist.” [ID 1820]
Patient-reported symptoms	Patient initiates contact with clinician and reports symptoms/seeks treatment.	“I forgot to document telephone encounter… I since have added details to her chart. Pt had been sick for 5–6 days with cough following flu like symptoms… I offered to send in medication without being seen due to extreme weather.” [ID 1790]

## Data Availability

Restrictions apply to the availability of these data as they are identifiable, protected health information of Northwestern Medicine patients. Making the data externally available would require significant approvals from the Northwestern Data Stewards and anonymization efforts.
